# Irisin attenuates type 1 diabetic cardiomyopathy by anti-ferroptosis via SIRT1-mediated deacetylation of p53

**DOI:** 10.1186/s12933-024-02183-5

**Published:** 2024-04-02

**Authors:** Yuan-juan Tang, Zhen Zhang, Tong Yan, Ken Chen, Guo-fan Xu, Shi-qiang Xiong, Dai-qian Wu, Jie Chen, Pedro A. Jose, Chun-yu Zeng, Jin-juan Fu

**Affiliations:** 1 Department of Cardiology, The Third People’s Hospital of Chengdu, Affiliated Hospital of Southwest Jiaotong University, Chengdu, 610031 China; 2 Department of Cardiology, Daping Hospital, Third Military Medical University (Army Medical University), Chongqing, 400042 China; 3 Key Laboratory of Geriatric Cardiovascular and Cerebrovascular Disease Research, Ministry of Education of China; Chongqing Key Laboratory for Hypertension Research, Chongqing Cardiovascular Clinical Research Center, Chongqing Institute of Cardiology, Chongqing, 400042 China; 4 Department of Cardiology and Endocrinolgy, Pangang Group Chengdu Hospital, Chengdu, 610066 China; 5 Department of Cardiovascular Surgery, The Third People’s Hospital of Chengdu, Affiliated Hospital of Southwest Jiaotong University, Chengdu, 610031 China; 6 Division of Renal Diseases & Hypertension, Department of Medicine and Department of Physiology/Pharmacology, The George Washington University School of Medicine & Health Sciences, Washington, DC 20037 USA; 7 State Key Laboratory of Trauma, Burns and Combined Injury, Daping Hospital, The Third Military Medical University, Chongqing, 400042 China; 8 Cardiovascular Research Center of Chongqing College, University of Chinese Academy of Sciences, Chongqing, 400042 China

**Keywords:** Type 1 diabetic cardiomyopathy, Ferroptosis, Irisin, p53, SIRT1

## Abstract

**Background:**

Diabetic cardiomyopathy (DCM) is a serious complication in patients with type 1 diabetes mellitus (T1DM), which still lacks adequate therapy. Irisin, a cleavage peptide off fibronectin type III domain-containing 5, has been shown to preserve cardiac function in cardiac ischemia–reperfusion injury. Whether or not irisin plays a cardioprotective role in DCM is not known.

**Methods and results:**

T1DM was induced by multiple low-dose intraperitoneal injections of streptozotocin (STZ). Our current study showed that irisin expression/level was lower in the heart and serum of mice with STZ-induced TIDM. Irisin supplementation by intraperitoneal injection improved the impaired cardiac function in mice with DCM, which was ascribed to the inhibition of ferroptosis, because the increased ferroptosis, associated with increased cardiac malondialdehyde (MDA), decreased reduced glutathione (GSH) and protein expressions of solute carrier family 7 member 11 (SLC7A11) and glutathione peroxidase 4 (GPX4), was ameliorated by irisin. In the presence of erastin, a ferroptosis inducer, the irisin-mediated protective effects were blocked. Mechanistically, irisin treatment increased Sirtuin 1 (SIRT1) and decreased p53 K382 acetylation, which decreased p53 protein expression by increasing its degradation, consequently upregulated SLC7A11 and GPX4 expressions. Thus, irisin-mediated reduction in p53 decreases ferroptosis and protects cardiomyocytes against injury due to high glucose.

**Conclusion:**

This study demonstrated that irisin could improve cardiac function by suppressing ferroptosis in T1DM via the SIRT1-p53-SLC7A11/GPX4 pathway. Irisin may be a therapeutic approach in the management of T1DM-induced cardiomyopathy.

**Supplementary Information:**

The online version contains supplementary material available at 10.1186/s12933-024-02183-5.

## Introduction

Diabetes is a non-communicable chronic metabolic disease that threatens global health, with type 1 diabetesmellitus (T1DM) accounting for approximately 2% of all diabetes cases [1, 2]. Diabetic cardiomyopathy (DCM) is a serious complication of T1DM, characterized by diastolic and systolic dysfunction, and is one of the most serious and common causes of morbidity and mortality in patients with T1DM [3]. Preclinical and clinical DCM has been extensively studied over the past several decades, however, adequate therapy is still lack until now.

The pathogenesis of DCM is multifaceted. Accumulating evidence suggests that the onset of cell death promotes the progression of diabetes-induced cardiac dysfunction. Although evidence suggests that apoptosis is the major type of cell death observed during DCM development [4]. Nevertheless, inhibiting apoptosis can only partly improve the survival rate of cardiomyocytes treated with high glucose (HG) [5]. Whether other non-apoptotic forms of regulated cell death also play a critical role during DCM development, especially in T1DM, is largely unknown. Ferroptosis is a new form of regulated cell death that is characterized by intracellular iron accumulation and Fe^2+^-dependent lipid peroxidation [6–8]. It is morphologically, biochemically, and genetically distinct from other types of cell death (apoptosis, autophagy, necrosis, and pyroptosis) [9]. However, the potential effect and regulatory mechanisms of ferroptosis on DCM were not exactly clarified. Only several recent findings suggest that ferroptosis is closely related to DCM onset [10, 11]. For example, Wang et al. found that ferroptosis was confirmed in the heart of type 2 diabetic mice, and inhibition of ferroptosis by liproxstatin-1 prevented the development of diastolic dysfunction [10]. Another work was performed in the setting of myocardial ischemia/reperfusion (MI/R) injury in streptozotocin-induced T1DM rats. The results indicated that ferroptosis is increased in DCM hearts relative to non-diabetic hearts. Inhibition of ferroptosis by ferrostatin-1 ameliorates in MI/R injury in DCM rats [12]. Therefore, the role of ferroptosis in DCM, especially in T1DM, remains poorly understood.

Exercise training confers sustainable protection against DCM ischemia/reperfusion injury in animal models [13]. Several myokines are produced by skeletal muscle, and changes in myokine levels following DCM were investigated [14–16]. Irisin, a cleavage peptideof fibronectin type III domain-containing protein 5 (FNDC5) [17], is highly expressed and released from cardiomyocytes [18, 19]. In addition to its effect on glucose and lipid homeostasis [20], it has therapeutic effects on cardiovascular disease, including atherosclerosis, cardiac hypertrophy, and cardiac ischemia–reperfusion injury [21]. Whether irisin plays a cardiac protective role in T1DM-induced cardiomyopathy is not known. Recently, several researches have shown that irisin could alleviate ferroptosis. For example, irisin protects against sepsis-associated encephalopathy by suppressing hippocampus ferroptosis via activating the nuclear factor erythroid-2 related factor 2 (NRF2) signaling pathway [22]. In addition, irisin protects against lung ischemia/reperfusion injury by anti-ferroptosis effects [23]. Whether irisin plays a cardiac protective role via against ferroptosis in DCM is unclear and demands further concerns. Therefore, we aim to verify the role of ferroptosis in the pathogenesis in DCM. Besides, whether irisin attenuates cardiac dysfunction by anti-ferroptosis effects in DCM and its underlying mechanisms are also investigated.

## Methods

### Reagents

Streptozotocin (STZ) (Sigma-Aldrich, St. Louis, MO) was dissolved in 0.1 mol/L citrate buffer (pH 4.5) for experiments in vivo*.* Irisin (Phoenix Pharmaceuticals, Burlingame, CA) was dissolved in saline. Erastin was obtained from Aladdin (Shanghai, China), cell counting kit-8 (CCK-8) kit, and EX527 (Sirt 1 inhibitor) were obtained from MCE (Shanghai, China). The assay kits for TNF-α, LI-1β, and IL-6 were purchased from SenBeiJia (Nanjing, China). The assay kits for MDA and LDH were purchased from Nanjing Jiancheng Bioengineering Institute (Nanjing, China). Cardiac lipid peroxidation (LPO) kit was purchased from Shanghai Enzyme-linked Biotechnology Co., Ltd. (Shanghai, China). GSH and GSSG kits, Western cell lysates, BCA protein assay kit, and cycloheximide (CHX) were purchased from Beyotime (Jiangsu, China). FerroOrange was purchased from the Maokang Institute of Biotechnology (Shanghai, China). Rabbit anti-irisin antibody was purchased from Phoenix Pharmaceuticals (Burlingame, CA). Rabbit anti-SLC7A11 antibody was purchased from Invitrogen (Carlsbad, CA). Rabbit anti-GPX4 antibody, mouse anti-tumor suppressor p53 (p53) antibody, rabbit anti-NRF2 antibody, and mouse anti-GAPDH antibody were purchased from Proteintech (Wuhan, China). Rabbit anti-SIRT1 antibody and rabbit anti- BRCA1 associated protein 1 (BAP-1) antibody were purchased from Affinity Biosciences (Jiangsu, China). Mouse anti-activating transcription factor 3 (ATF3) antibody was purchased from Santa Cruz Biotechnology (Dallas, TX). Rabbit anti-acK382-p53 was purchased from Cell Signaling Technology (Beverly, MA). IRDye 800CW donkey anti-rabbit antibody and anti-mouse antibody were purchased from Li-Cor Biosciences (Bad Homburg, Germany).

### Animals

Eight-week-old C57BL/6 J mice, purchased from Beijing Vital River Laboratory Animal Technology *Co., Ltd.* (Beijing, China), were kept at room temperature (~ 23 ℃) under a standard 12:12 h (hrs) light/dark cycle. The mice were intraperitoneally injected with freshly prepared STZ at the dose of 50 mg/kg body weight for 5 consecutive days to induce T1DM models, as described previously [24]. Two weeks after the last injection of STZ, the mice were fasted for 12 h before measuring blood glucose (tail vein). Diabetes mellitus was confirmed as the fasting blood glucose levels were more than 16.7 mmol/L [25]. Mice that were intraperitoneally injected with the same volume of citrate buffer served as controls (vehicle). The blood glucose levels and body weight were monitored in weeks 0, 1, 3, 5, and 7. The blood samples for plasma glucose determination were taken from the tail vein. At the end of the study, the mice were euthanized with an overdose of sodium pentobarbital (100 mg/kg body weight, intraperitoneal injection), and the hearts were collected and stored for further studies.

All experiments conformed to the recommended guidelines for animal experimentation and were approved by the Animal Ethics Committee of Daping Hospital, Third Military Medical University (AMUWEC20219031), and these experiments conformed to the NIH guidelines for the care and use of laboratory animals.

### Echocardiography

Echocardiography was performed using a small-animal high-resolution ultrasound imaging system (Vevo 2100, Visual Sonics, Canada). The mice were anesthetized with 2% isoflurane (Baxter) and the heart rates were 450–550 beats/min [26]. Two-dimensional guided M-mode measurements at the parasternal long-axis plane were performed for at least two beats and then averaged. The left ventricle internal diameter at end-diastole (LVIDd) and end-systole (LVIDs) were measured. Left ventricular ejection fraction (LVEF) and left ventricular fractional shortening (LVFS) were calculated with the supporting software packages (Visual Sonics, Canada).

### Enzyme-linked immunosorbent assay

Mice were anesthetized with sodium pentobarbital and after one hour, blood samples (500 μL) were collected from the posterior orbital sinus. The blood samples were centrifuged at 3000 × *g* for 20 min, and the serum was collected for further analysis. The concentrations of serum TNF-α, IL-1β, and IL-6 were measured using ELISA kits.

### Histological evaluation

The hearts were rapidly washed with pre-cooled Phosphate-buffered saline (PBS) at 4 ℃ and fixed overnight with 4% paraformaldehyde. The samples were then dehydrated with gradient alcohol and embedded in paraffin followed by serial slicing of 4 µm sections. The heart sections were stained with hematoxylin and eosin (HE, Solarbio Life Science, Beijing, China), according to standard protocols. The sections were analyzed by the M8 Digital Scanning Microscopy System (PreciPoint, Freising, Germany). To quantify the fibrosis, the heart sections were stained with Masson’s trichrome (Masson) stain kit (Solarbio Life Science). According to the kit’s instructions, the sections were treated with hematoxylin for 10 min, ferric oxide for 5 min, acid fuchsin for 10 min, phosphomolybdate for 10 min, and acetic acid for 1 min. The myocardial collagen area was quantified by measuring the ratio of fibrosis in the stained area to the total stained areausing ImageJ. Six randomly selected microscope fields (× 400) were used for histological analysis.

### Prussian Blue staining

Iron accumulation was measured by Prussian Blue staining (Solarbio Life Science). Briefly, the heart sections were deparaffinized at 65 ℃ and rehydrated in double distilled water. Prussian Blue staining of the heart sections was performed using freshly prepared equivalent volumes of potassium ferrocyanide solution and hydrochloric acid solution. After ten minutes the sections were rinsed in distilled water and counterstained with nuclear fast red, made transparent, and cover-slipped. Finally, the sections were analyzed by the software viewpoint M8 Digital Scanning Microscopy System (PreciPoint, Freising, Germany). Iron deposition (blue) in six images from each section was analyzed by Image J (× 400).

### Transmission electron microscopy

Transmission electron microscopy was used to analyze the mitochondrial damage in the heart, according to a published protocol. Briefly, the heart sections were washed with pre-cooled PBS at 4 ℃ and then fixed with 2% (w/v) glutaraldehyde buffer overnight at 4 ℃. Then the samples were fixed with 0.1 mol/L phosphate-buffered saline (pH 7.4), containing 1% osmic acid for 2 h at room temperature, and then washed three times in cold 0.1 mol/L phosphate buffer (pH 7.4) for 15 min. Subsequently, the samples were dehydrated with a series of alcohol concentrations and embedded in epon-araldite resin. Ultrathin sections were obtained by an ultramicrotome, followed by staining with uranyl acetate and lead citrate and imaging with a JEM-1400Plus electron microscope (Hitachi, Tokyo, Japan). The ultrastructural damage of mitochondria was rated on a scale of 0 to 4 in severity [27]: normal structure with well-preserved mitochondrial granules = 0; deleted mitochondrial granules = 1; swollen mitochondria with clearing of the matrix = 2; 3 = disruption of mitochondrial crests with clearing as well as condensation of the matrix = 3; 4 = disruption of the crests and the mitochondrial inner and outer membrane = 4. The score for each mouse was calculated as the average score of six heart slices.

### In vitro experiments

The immortalized rat cardiomyocyte cell line (H9C2) was purchased from the American Type Culture Collection (ATCC, Manassas, VA) and cultured in Dulbecco’s Modified Eagle Medium (DMEM, Gibco; Gaithersburg, MD) containing 5.5 mmol/L glucose, supplemented with 10% fetal bovine serum (FBS; Gibco) and 100 U/mL penicillin/streptomycin (Beyotime, Jiangsu, China) in a humidified incubator with 5% CO_2_ at 37 ℃. To establish a DCM model in vitro, H9C2 cells were cultured in 1% FBS-supplemented DMEM containing 5.5 mmol/L glucose for 16 h and then in high glucose concentration (35 mmol/L) for 24 h [28].

### Transfections experiments

Cloning Vector pcDNA3.1 ( +) (Vector)was used to construct the p53 overexpression sequence (Hanheng Biology, Shanghai, China). Lipofectamine 8000 (Beyotime) was used for transient transfection of Vector and p53 plasmids into H9C2 cells, following the manufacturer’s protocol. To silence SIRT1, the H9C2 cells were transfected SIRT1 siRNA (50 nmol/L) for 48 h using lipofectamine RNA imax (Invitrogen, Carlsbad, CA), according to the instructions provided by the manufacturer. Non-silencing SIRT1 RNA served as the negative control (NC, 50 nmol/L), SIRT1 siRNA was purchased from Ribobio (Guangdong, China). The sequence of SIRT1 (rat) siRNAs is: 5ʹ-GCCACCAACACCTCTTCAT -3ʹ.

### Cell viability determination

Cell viability was measured using a CCK8 Kit, according to the manufacturer’s protocol. Briefly, when H9C2 cells in 96-well plates reached sub-confluence (70–80%), they were treated with varying concentrations of irisin or vehicle (sterilized PBS) for 24 h. Then 10 µl CCK-8 solution was added into each well for another 60 min at 37 ℃ in the dark. The microplate reader (ThermoFisher Scientific) was used to measure the absorbance (optical density, OD) at 450 nm.

Lactate dehydrogenase (LDH) levels in the H9C2 cells were measured using commercial kits (Nanjing Jiancheng Bioengineering Institute, Nanjing, China), according to the manufacturer’s protocol. Briefly, cell culture medium supernatants were collected and LDH assay reagents were added for 30 min. OD of each well was measured by a microplate reader at 440 nm.

### Determination of ROS generation

To quantify the levels of reactive oxygen species (ROS) in H9C2 cells, the cells were treated as indicated and then incubated with 10 µmol/L 2, 7-dichlorofluorescein diacetate (DCFH-DA, Beyotime, Jiangsu, China) for 30 min at 37 ℃ in the dark. Green fluorescence was detected using a confocal laser scanning microscope (Olympus, Tokyo, Japan). The fluorescent intensity in different groups was determined by Image J.

### Determination of lipid peroxidation

To measure lipid peroxidation levels in cardiac tissue lysates and H9C2 cells, they were homogenized in ice-cold lysis buffer containing protease inhibitor cocktails [29]. The supernatant was collected by centrifugation (1200 *g*, 4 ℃, 10 min). MDA levels were measured using a lipid peroxidation MDA assay kit. Cardiac LPO levels were measured and calculated according to the manufacturer’s protocol. The standard, blank, and sample wells were assayed individually, and the absorbance at 450 nm was recorded. Reduced glutathione (GSH) and oxidized glutathione (GSSG) levels were measured using the Glutathione Fluorometric Assay Kit. Briefly, the hearts and H9C2 cells were ground in a protein removal reagent M solution. The homogenates were centrifuged at 10,000 × *g* for 10 min at 4 ℃, and the supernatant was collected. The levels were measured according to the instructions in the kit.

### Western blot

The heart and H9C2 cell proteins were extracted with western cell lysis, containing protease inhibitor cocktails (Roche, Indianapolis, IN). The protein concentrations were measured by the BCA protein assay kit. Briefly, the same amounts of denatured protein samples (60 µg) were separated by 10% sodium dodecyl sulfate–polyacrylamide gel electrophoresis (SDS-PAGE) and then electrophoretically transferred onto nitrocellulose filter membranes (Bio-Rad, Hercules CA). After the nonspecific binding sites were blocked in tris-buffered saline (TBS) containing 5% nonfat dry milk for 1 h, the blots were washed three times with TBS containing Tween 20 (TBST). The membranes were incubated with the primary antibodies at 4 ℃ overnight and then washed three times with TBST, before their incubation with IRDye 800CW donkey anti-rabbit antibody (1:10,000) or anti-mouse antibody (1:10,000) for 1 h at room temperature, followed by three washings with TBST. Finally, the bands were visualized by the Odyssey Western blot Detection System (Li-Cor Biosciences; Lincoln, NE). The images were analyzed using the Odyssey Application Software to obtain integrated intensities. The membranes were immunoblotted with the following primary antibodies: rabbit anti-irisin (1:500), rabbit anti-SLC7A11 (1:1000), rabbit anti-GPX4 (1:1000), mouse anti-p53 (1:1000), rabbit anti-acK382-p53 (1:1000), rabbit anti-SIRT1 (1:500), rabbit anti-NRF2 (1:1000), mouse anti-ATF3 (1:500), rabbit anti-BAP-1 (1:500), and mouse anti-GAPDH (1:40,000).

### Quantitative real‑time PCR

The procedure for RNA isolation and quantitative real-time polymerase chain reaction (qRT-PCR) analysis has been previously described [30]. In brief, total RNA was extracted from H9C2 cells using TRIzol Reagent. Subsequently, the RNA concentration was measured using a NanoDrop 2000 spectrophotometer (Thermo Fisher Scientific), and cDNA was synthesized from 1 μg of RNA using the PrimeScript RT Master Mix (Takara) through the reverse transcription system. 1 μg total cDNA was subjected to qRT-PCR using the Bulge-Loop miRNA qRT-PCR Starter Kit (Ribobio) according to the instructions. Then, the designed GAPDH, p53 primer sequence (additional file 1: Table S1) is based on NCBI (https://www.ncbi.nlm.nih. gov/tools/prime-blast/). The detected endogenous genes were normalized using GAPDH as an internal control.

### Intracellular iron assay

FerroOrange was used to measure the levels of cellular Fe^2+^. H9C2 cells were seeded at 1 × 10^5^ per well in 24-well plates with coverslips. After the indicated treatment, the H9C2 cells were incubated in a complete DMEM medium with FerroOrange (1 μmol/L) for 30 min in the dark. The fluorescence images were acquired with a laser confocal microscope at 555 nm laser line (Olympus, Tokyo, Japan) and the fluorescence intensity was calculated by Image J. The results were acquired from at least six different fields of sections of the heart (× 400).

### Statistical analysis

All quantitative values are presented as mean ± standard deviation (SD). Mapping and analyses were performed using GraphPad Prism 8 (San Diego, CA) and SPSS 25 software (Chicago, IL), respectively. Student’s t-test was used to compare the data from two groups, and one-way analysis of variance (ANOVA) with Bonferroni’s post-hoc test was used to compare the data from three or more groups, after checking for normality (Kolmogorov–Smirnov) and homogeneity (Levene). Statistical significance was set at *P* < 0.05.

## Results

### Irisin supplementation improves cardiac function in STZ-induced type 1 diabetes

**Fig. 1:**
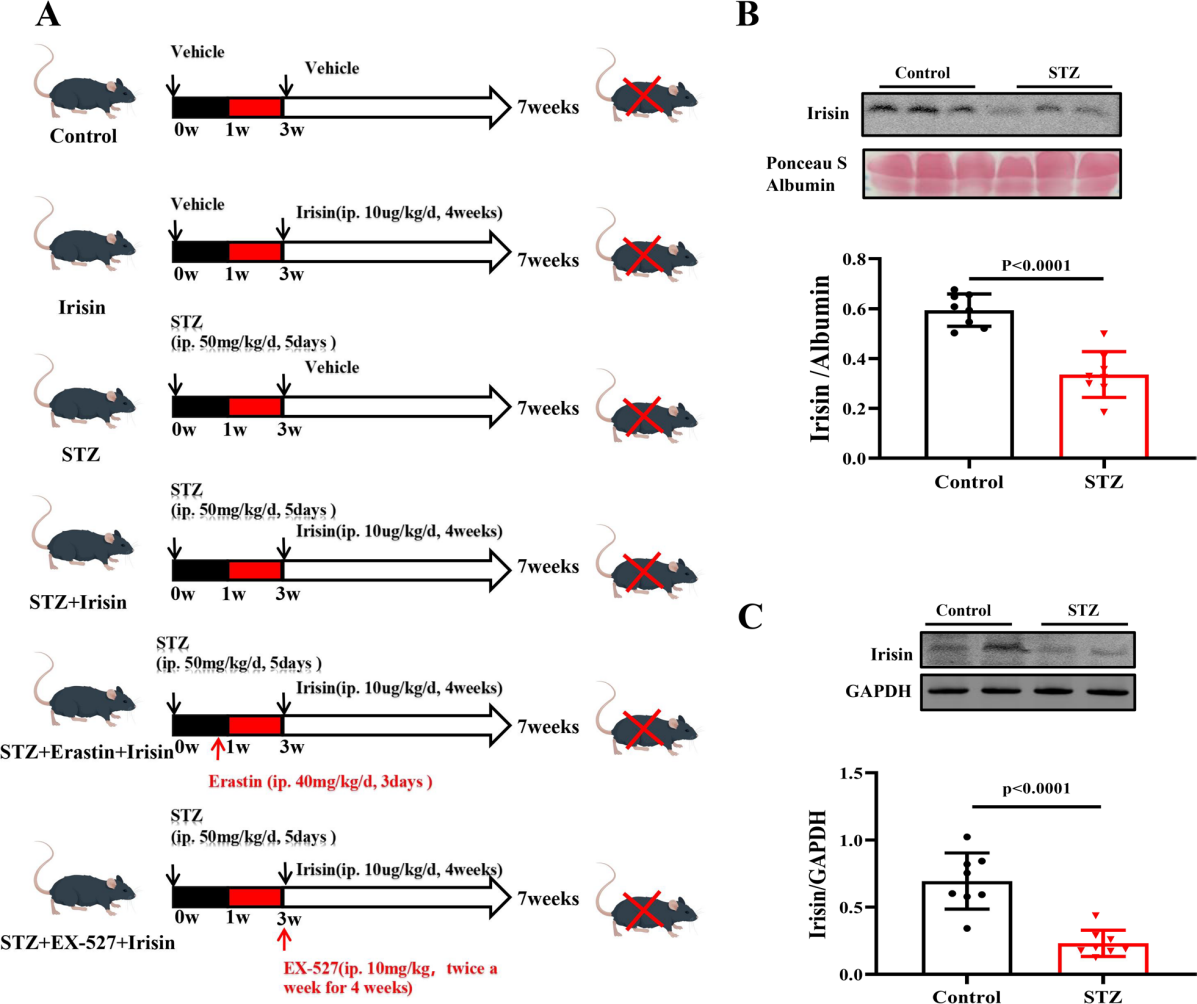
The heart tissue and serum levels of irisin are decreased in type 1 diabetic mice. **A** A schematic diagram showing the treatments of the mice. **B** Western blot of serum samples obtained from type 1 diabetic mice and control non-diabetic mice (10 μL serum per lane). Ponceau S staining Albumin served as loading control (n = 8 per group). **C** Irisin protein expression in the heart was determined by Western blot. GAPDH served as the loading control. Data are expressed as the mean ± SD. Student’s t-test.^*^P < 0.05, ^**^P < 0.01. *STZ* streptozotocin

**Fig. 2:**
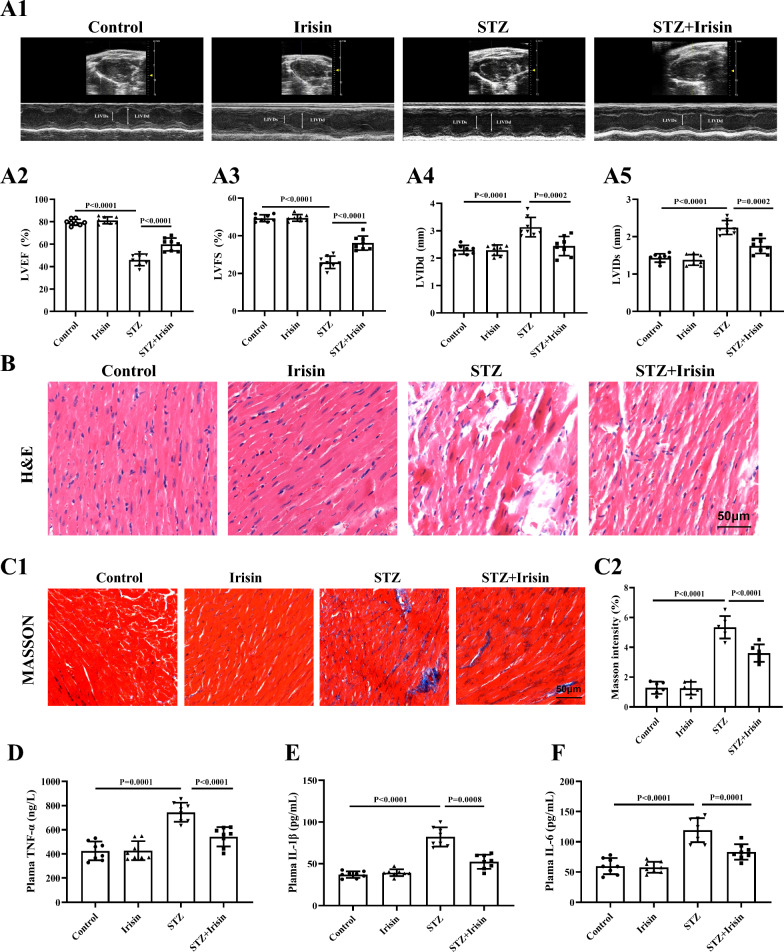
Irisin improves cardiac function and ameliorates cardiac remodeling in type 1 diabetic mice. The mice were treated with STZ for 5 days to induce diabetic cardiomyopathy; similarly managed mice injected vehicle without STZ served as controls. Then the control and diabetic mice were intraperitoneally injected with irisin (10 μg/kg body weight/day) or vehicle for 4 consecutive weeks. **A** Representative M-mode echocardiography (**A1**) from a mouse in each of the four groups of mice. Quantification of LVEF% (**A2**), LVFS% (**A3**), LVIDd (**A4**), and LVIDs (**A5**) in the indicated groups (n = 8 per group). **B** The histopathological changes of myocardial tissues using H&E staining from a mouse in each of the four groups of mice (Scale bar = 50 μm; n = 6 per group). **C.** Representative Masson staining (**C1**) and quantification (**C2**) of the fibrotic area from a mouse in each of the four groups of mice (Scale bar = 50 μm; n = 6 per group). The serum levels of inflammation-associated biomarkers, such as TNF-α (**D**), IL-1β (**E**), and IL-6 (**F**) were shown (n = 8 per group). Data are presented as the mean ± SD.One-way ANOVA, and Bonferroni’s post-hoc test. ^*^P < 0.05, ^**^P < 0.01. *STZ* streptozotocin, *LVEF* left ventricular ejection fraction, *LVFS* left ventricular fractional shortening, *LVIDd* left ventricular internal diameter at end-diastole, and *LVIDs* left ventricular internal diameter at end-systolic, *H&E* hematoxylin and eosin, *TNF- α* tumor necrosis factor-α, *IL-1β* interleukin-1β, and* IL-6* interleukin-6

Successful establishment of TIDM by the intraperitoneal injection of STZ at the dose of 50 mg/kg body weight for 5 consecutive days in 8-week-old C57BL/6 J mice (Fig. 1A) was confirmed by elevated fasting serum glucose concentration (> 16.7 mmol/L). After that, we measured serum and myocardial irisin levels and found that irisin proteins were lower in serum and hearts from STZ-treated mice than control mice (Fig. 1B and C). Consistent with other reports [31], STZ-treated mice had impaired cardiac function, which was ameliorated (LVEF and LVFS) (Fig. 2A1, 2A2, and A3) or normalized (LVIDd and LVIDs) (Fig. 2A4 and 5) by irisin (10 μg/kg body weight/day), intraperitoneally injected daily for 4 weeks, although irisin did not affect the body weights or blood glucose levels of T1DM mice (Additional file 1: Figures S1A and B).

Consistent with other reports [32, 33], STZ-induced T1DM in mice displayed typical morphological and biochemical changes, including ruptured and disorganized myofibrils and cardiac fibrosis (Fig. 2B and C), and increased serum levels of inflammatory factors (TNF‐α, IL‐1β, and IL-6) (Fig. 2D–F). However, irisin treatment ameliorated or normalized the above-mentioned abnormal changes, including the morphological and biochemical parameters (Fig. 2B–F).

### Irisin ameliorates cardiac function in STZ-induced T1DM mice by reducing ferroptosis

**Fig. 3:**
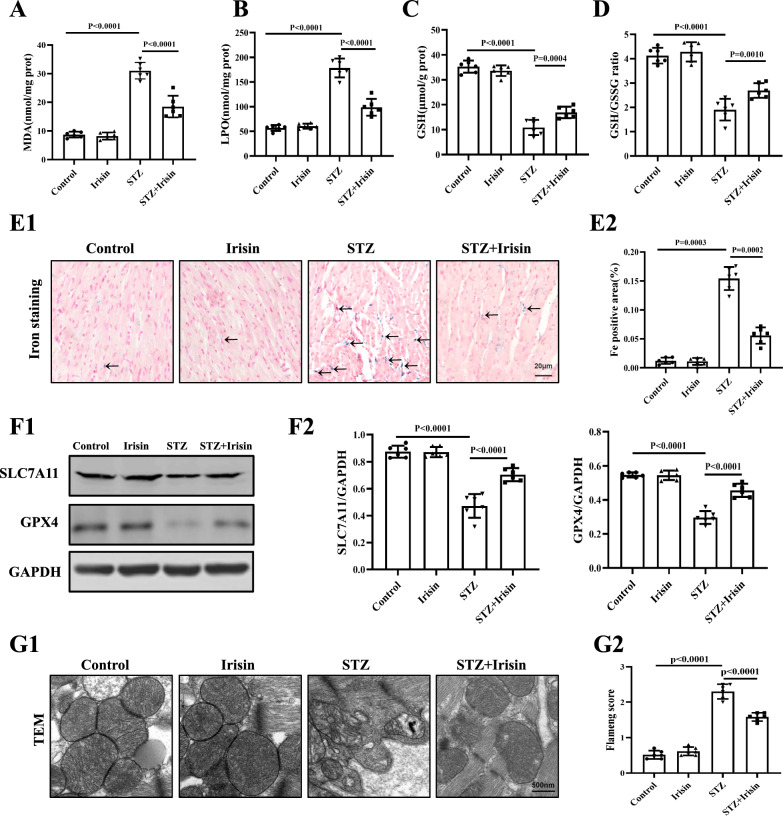
Irisin attenuates ferroptosis in the heart of type 1 diabetic mice. **A** MDA, **B** LPO, **C** GSH, and **D** GSH/GSSH ratio in cardiac tissue lysates were measured from the four groups of mice (n = 6 per group). **E** Representative images (**E1**) and the statistical results (**E2**) of the iron staining (Scale bars = 20 μm; n = 6 per group). **F** Representative Western blots (**F1**) and quantification (**F2**) showing myocardial protein levels of SLC7A11 and GPX4 in the four groups of mice (n = 6 per group). **G** Representative TEM images (**G1**) showing the mitochondria of cardiac tissue from a mouse in each of the four groups of mice (Scale bars = 500 nm) and (**G2**) The corresponding relative Flameng scores were shown (n = 6 per group). Data are expressed as the mean ± SD.One-way ANOVA, and Bonferroni’s post-hoc test. *P < 0.05, **P < 0.01. *STZ* streptozotocin, *MDA* malondialdehyde, *LPO* lipid peroxides, *GSH* reduced glutathione, *GSSG* oxidized glutathione, *TEM* transmission electron microscopy, *SLC7A11* solute carrier family 7 member 11, and *GPX4* glutathione peroxidase 4

Ferroptosis is an important pathological mechanism underlying DCM in mice with type 2 diabetes mellitus (T2DM) [10]. To determine whether ferroptosis is involved in the pathogenesis of T1DM, we first measured lipid peroxidation marker (LPO, MDA, and GSH) levels in heart tissue. As shown in Fig. 3A–D, STZ-induced T1DM in mice was accompanied by increased MDA and LPO levels and decreased GSH levels and GSH/GSSG ratios, which were ameliorated by irisin but had no effect in control mice. Moreover, we found that STZ-induced diabetic heart had higher staining of iron deposition (Fig. 3E) and decreased expressions of essential ferroptosis-related proteins (SLC7A11 and GPX4) (Fig. 3F), that were also recovered by irisin. The mitochondria are very important in the protection against ferroptosis [34]. Transmission electron microscopy (TEM) analysis showed shrunken mitochondria with enhanced membrane density, a typical morphological feature of ferroptosis [8]; irisin treatment ameliorated these mitochondrial abnormalities (Fig. 3G).

**Fig. 4:**
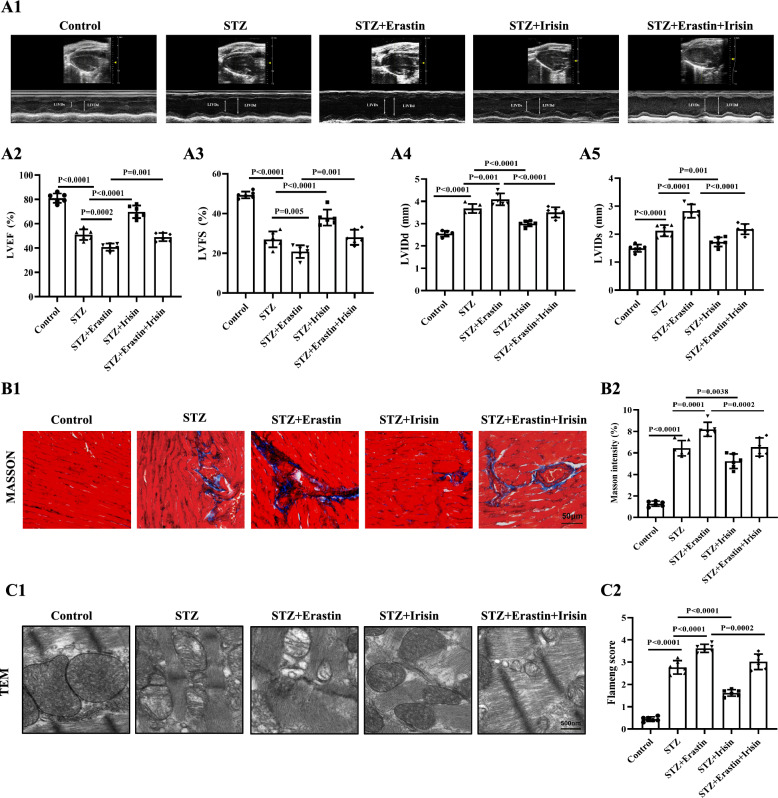
Erastin abolishes the protective effect of irisin on diabetic cardiomyopathy. Type 1 diabetic mice were treated with irisin (10 μg/kg body weight/day) in the presence or absence of the ferroptosis inducer erastin (40 mg/kg body weight/day, 3 consecutive days for 4 weeks). A1. Representative echocardiographic images from a mouse in each of the five groups of mice. Quantification of (**A2**) LVEF%, (**A3**) LVFS%, (**A4**) LVIDd, and (**A5**) LVIDs are shown (n = 6 per group). **B** Representative Masson staining images of heart tissues from a mouse in each of the five groups of mice: (**B1**) quantification (**B2**) of the fibrotic area (Scale bar = 50 μm; n = 6 per group). **C** Representative TEM images from the heart of a mouse in each of the five groups of mice (**C1**) and corresponding relative Flameng scores (Bottom) (**C2**) of the mitochondria in myocardial tissues of each group (Scale bars = 500 nm; n = 6 per group). Data are presented as the mean ± SD. One-way ANOVA, and Bonferroni’s post-hoc test. ^*^P < 0.05, ^**^P < 0.01. *STZ* streptozotocin, *LVEF* left ventricular ejection fraction, *LVFS* left ventricular fractional shortening, *LVIDd* left ventricular internal diameter at end-diastole, and *LVIDs* left ventricular internal diameter at end-systole, *TEM* transmission electron microscopy

To further verify the role of ferroptosis on irisin-mediated protection of DCM in T1DM in mice, we administered the ferroptosis inducer erastin (40 mg/kg body weight/day, for 3 consecutive days) to STZ-induced diabetic mice [35]. The protective effects of irisin on the heart of STZ-induced diabetic mice included improvement in cardiac function, mitigation of pathological structure disorder and fibrosis, and restoration of mitochondrial structure in cardiomyocytes. However, STZ supplementation with erastin exacerbated heart injury, and erastin mitigated the above-mentioned protective effect of irisin (Fig. 4). These results suggest that the targeting of ferroptosis is responsible for the cardiac protective effects of irisin in DCM.

### Irisin down-regulates p53 to promote the expression of SLC7A11/GPX4 and consequently inhibits ferroptosis

**Fig. 5:**
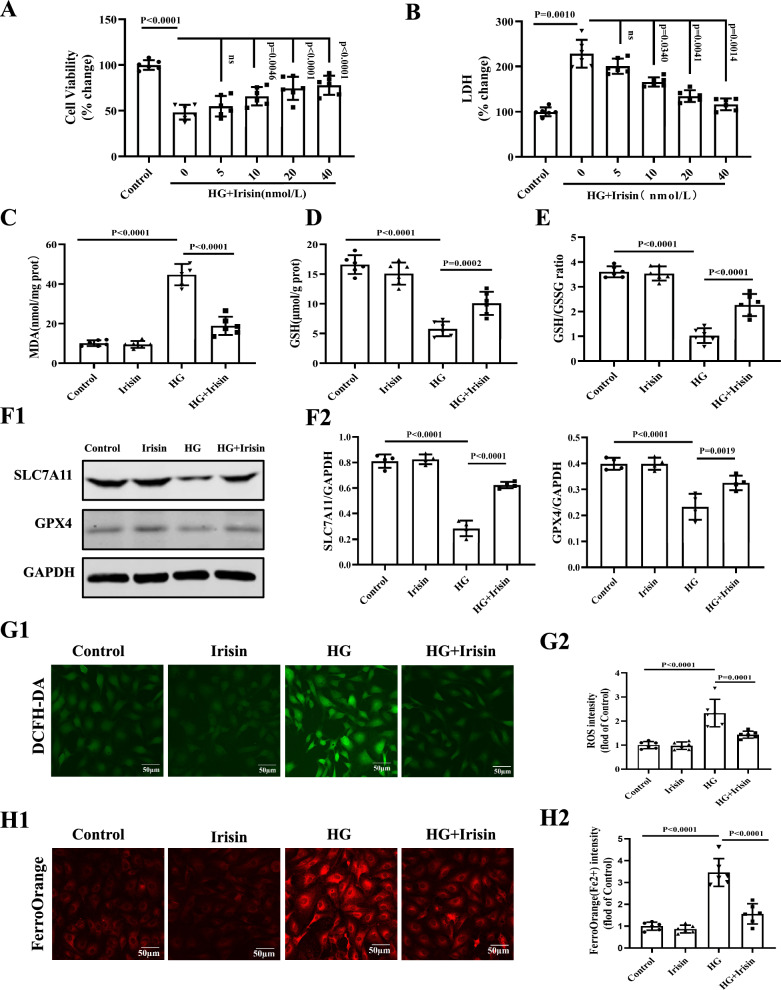
Irisin inhibits HG-induced cell damage and ferroptosis in H9C2 cells. H9C2 cells were incubated with HG (35 mmol/L) for 24 h in the presence or absence of irisin (10–40 nmol/L) before physiological/biochemical assessment. The cell viability of H9C2 cells was determined by the CCK-8 Kit (**A**) and LDH release assay (**B**) (n = 6 per group). MDA (**C**), GSH (**D**), and GSH/GSSG ratio (**E**) in H9C2 cells were determined using the relevant kits (n = 6 per group). **F** Western blots of the proteins SLC7A11 and GPX4 in H9C2 cells. Representative blots from a mouse in each of the four groups of mice (**F1**) and quantitative analysis of SLC7A11 and GPX4 (**F2**) are shown (n = 4 per group). GAPDH served as the loading control. **G** Representative images of fluorescence probe for ROS from H9C2 cells in each of the four groups of H9C2 cells (**G1**) with quantification (**G2**) (Scale bar = 50 μm; n = 6 per group).** H1** Representative fluorescent images of FerroOrange staining in H9C2 cells in each of the four groups of H9C2 cells (Scale bar = 50 μm).** H2** The quantitative results of FerroOrange staining are shown (n = 6 per group). Data are presented as the mean ± SD.One-way ANOVA, and Bonferroni’s post-hoc test. ^*^P < 0.05, ^**^P < 0.01. *HG* high glucose, *MDA* malondialdehyde, *GSH* reduced glutathione, *GSSG* oxidized glutathione, *SLC7A11* solute carrier family 7 member 11, and *GPX4* glutathione peroxidase 4, and *LDH* lactate dehydrogenase

To investigate the underlying mechanism(s) of the irisin-mediated cardiac protection, we studied the role of ferroptosis in H9C2 cells treated with HG (35 mmol/L). HG decreased H9c2 cell viability and increased LDH concentration in the culture medium, which was reversed or ameliorated by irisin in a concentration (5–40 nmol/L) -dependent manner (Fig. 5A and B). HG increased MDA levels, decreased GSH, GSH/GSSG ratio, SLC7A11, and GPX4 expressions, accompanied by increased ROS levels. FerroOrange fluorescent probes also showed that the intracellular Fe^2+^ levels were increased by HG. Irisin (10 nmol/L) treatment ameliorated the above-mentioned changes in H9C2 cells (Fig. 5C–H).

**Fig. 6:**
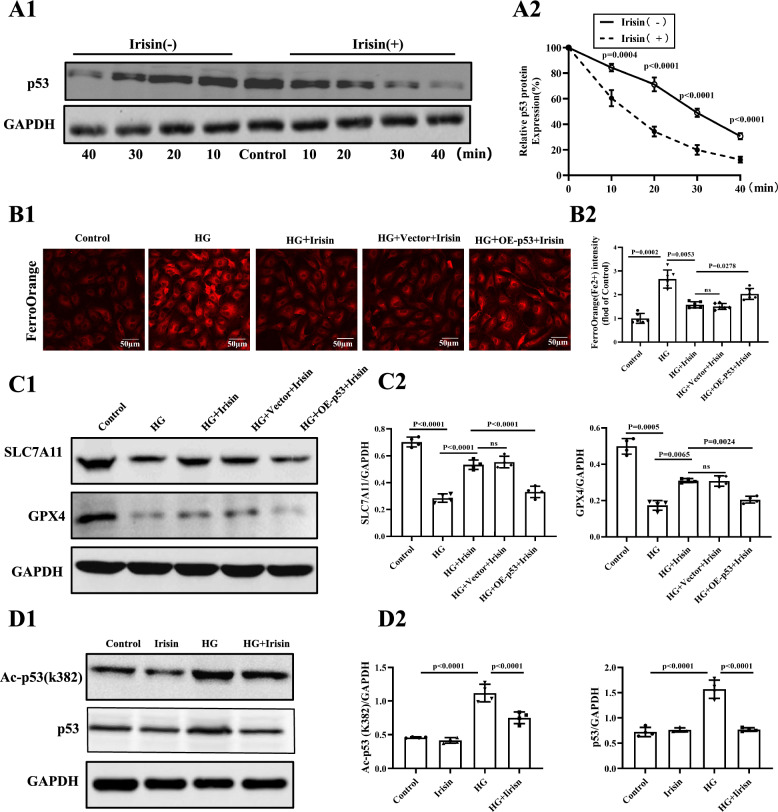
Irisin down-regulates p53 to promote the expression of SLC7A11 and consequently inhibits ferroptosis in H9C2 cells. **A** The effect of the irisin on the time course of p53 degradation in HG-treated H9C2 cells. To inhibit protein synthesis, the cells were treated with 100 μmol/L CHX for the indicated time (n = 4 per group). **B** Representative fluorescent images of FerroOrange staining in each of the four groups of H9C2 cells (**B1**) and the quantification (**B2**) in H9C2 cells (Scale bar = 50 μm; n = 6).** C1** Immunoblots of SLC7A11, GPX4, and GAPDH from each of the five groups of H9C2 cells.** C2** Quantification of the SLC7A11 and GPX4 bands. All results were normalized to the expression level of GAPDH (n = 4 per group). **D** Representative Western blotting images (**D1**) with quantification (**D2**) from each of the five groups of H9C2 cells showing p53 K382 acetylation andp53 protein levels (n = 4 per group). Data are expressed as the mean ± SD. One-way ANOVA, and Bonferroni’s post-hoc test. ^*^P < 0.05, ^**^P < 0.01. *HG* high glucose, *p53* tumor suppressor p53, *SLC7A11* solute carrier family 7 member 11, and *GPX4* glutathione peroxidase 4, *CHX* cycloheximide, and *Ac* acetylation

It is well documented that SLC7A11 and GPX4 play a vital role in the signal transduction of ferroptosis, which function as importers of cystine and synthesizers of GSH to reduce lipid peroxidation and suppress ferroptosis [36, 37]. Previous studies have shown that the upstream regulators of SLC7A11include p53, NRF2, ATF3, and BAP-1 [38–41]. After screening for these proteins, we found that p53 was the only possible candidate, i.e., HG increased p53 expression, which was reduced by irisin (Additional file 1: Figure S2A). Consistent with this finding, western blotting results showed increased p53 protein expression in the hearts of the T1DM mice compared with the control groups, which was partially repressed by irisin treatment (Additional file 1: Figure S2B). Interestingly, irisinn did not affect p53 mRNA expression in HG-treated H9C2 cells (Additional file 1: Figure S2C), indicating that the irisin-mediated effect on p53 expression in HG-treated H9C2 cells could be due to post-transcriptional regulation. Indeed, our further experiments showed that irisin treatment profoundly promoted p53 protein degradation in the CHX (100 µmol/L) chase experiment. The half-life of p53 was shortened from 30 min in control conditions to 15 min in irisin-stimulated cells (Fig. 6A). The ability of p53 to interfere with the protective effect of irisin on ferroptosis in H9C2 cells was shown further by the ability of p53 over-expression (Additional file 1: Figure S3A) to block the negative effect of irisin on ferroptosis, i.e., decrease in Fe^2+^ accumulation and increase in SLC7A11 and GPX4 expressions in HG-treated H9C2 cells (Fig. 6B and C), indicating that irisin inhibited ferroptosis by a p53-inhibitory-dependent manner. It is known that the stability of p53 protein is affected by its acetylation [42]. Our current study found that HG increased p53 activity, indicated by increased levels of acetylated p53; irisin treatment significantly reduced the acetylation of p53 at lysine K382 (Fig. 6D).

### Irisin attenuates STZ-induced diabetic cardiomyopathy through the SIRT1/p53 pathway

**Fig. 7:**
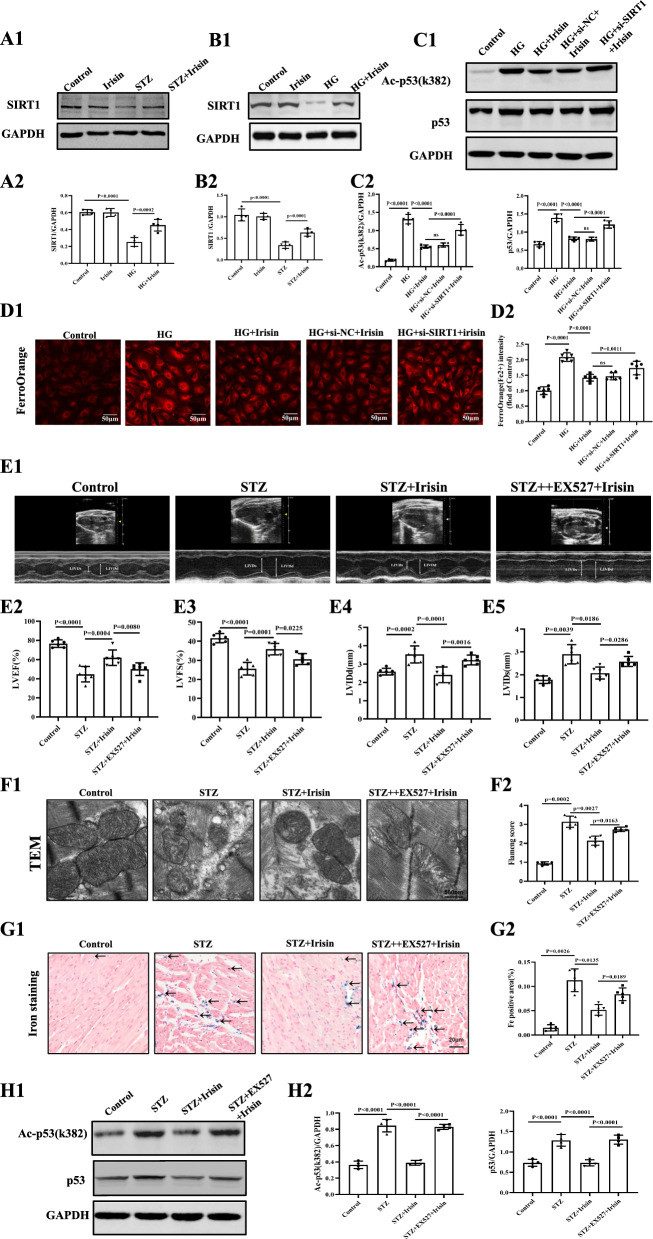
Irisin attenuates ferroptosis through the SIRT1/p53 Pathway. **A** SIRT1 protein expression in the heart was detected by western blotting (**A1**) and quantitative analysis (**A2**), All results were normalized to the expression level of GAPDH (n = 4 per group). **B** Representative Western blots in each of the four groups of H9C2 cells (**B1**) and quantification (**B2**) of the protein levels of SIRT1 in H9C2 cells, All results were normalized to the expression level of GAPDH (n = 4 per group). **C** Western blot images (**C1**) and quantification (**C2**) in each of the four groups of p53 K382 acetylation and p53 protein in H9C2 cells (n = 4 per group). **D1** Representative fluorescent images of FerroOrange staining in each of the five groups of H9C2 cells (scale bar = 50 μm) and (**D2**) quantification of the FerroOrange fluorescence intensity (n = 6 per group).** E1** Representative images of M-mode echocardiography of a mouse in each of the four groups of mice. Statistical analyses of (**E2**) LVEF%, (**E3**) LVFS%, (**E4**) LVIDd, and (**E5**) LVIDs in the indicated groups (n = 6 per group). **F** Representative TEM images of the heart tissue from a mouse in each of the four groups of mice (**F1**) and corresponding relative Flameng scores (Bottom) (**F2**) for mitochondria cardiac tissue (Scale bars = 500 nm; n = 5 per group). **G** Representative images of iron staining of the heart from a mouse in each of the four groups of mice (**G1**) and quantitative analysis (**G2**) of iron deposition in the four groups of mice (Scale bars = 20 μm; n = 5 per group). **H** p53 K382 acetylation levels and p53 protein levels were determined by Western blotting of the heart from a mouse in each of the four groups of mice (n = 4). Data are expressed as the mean ± SD. One-way ANOVA, and Bonferroni’s post-hoc test. ^*^P < 0.05, ^**^P < 0.01. *HG* high glucose, *SIRT1* Sirtuin 1, *STZ* streptozotocin, *LVEF* left ventricular ejection fraction, *LVFS* left ventricular fractional shortening, *LVIDd* left ventricular internal diameter at end-diastole, *LVIDs* left ventricular internal diameter at end-systole, and *TEM* transmission electron microscopy

**Fig. 8:**
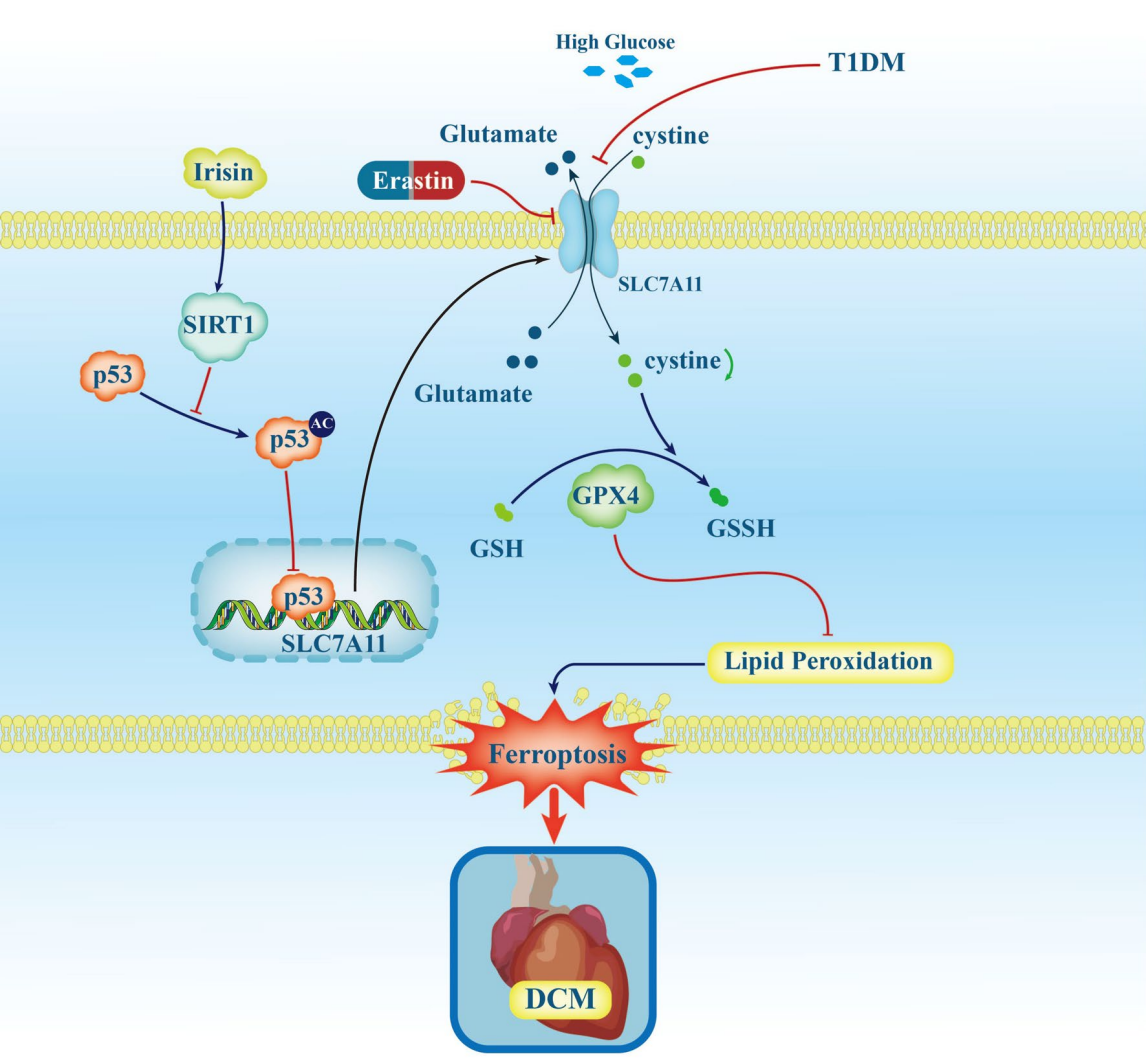
Schematic diagram summarizing the protective effects of irisin treatment in type 1 diabetic cardiomyopathy through anti-ferroptosis via induction of the SIRT1-p53-SLC7A11/GPX4 pathway. High glucose, a key pathogenic factor of DCM of the type 1 diabetic model, increases intracellular Fe^2+^ and lipid peroxidation, along with GSH depletion and SLC7A11(cystine/glutamate antiporter)/GPX4 inhibition, implying a role for ferroptosis in the pathogenesis of T1DM. Irisin suppresses ferroptosis and alleviates cardiac remodeling and dysfunction via activation of the SIRT1-p53-SLC7A11/GPX4 pathway. *DCM*, diabetic cardiomyopathy, *SLC7A11* solute carrier family 7 member 11, *GPX4* glutathione peroxidase 4, *Ac* acetylation, *GSH* reduced glutathione, and *SIRT1* Sirtuin 1

Next, we screened for possible candidate genes, which may be responsible for p53 acetylation. SIRT1 struck our attention because activation of SIRT1 deacetylates and inactivates p53, thereby affecting its stability [43, 44]. Moreover, irisin, via the SIRT1/Nrf2 pathway,attenuates acute kidney injury in septic mice [45]. We found that the expression of SIRT1 was significantly decreased in STZ-treated mice than in control mice, which was significantly reversed by irisin treatment. (Fig. 7A). Besides, our results also showed that HG down-regulated SIRT1 protein expression, which was almost completely reversed by irisin (Fig. 7B). Knocking down SIRT1 by siRNA (Additional file 1: Figure S3B) markedly abolished the irisin-mediated down regulation of p53 protein expression and p53 acetylation levels in HG-treated H9C2 cells (Fig. 7C). Consistent with the above results, SIRT1 knockdown also impaired the inhibitory effect of irisin on Fe^2+^ accumulation in HG-treated cells (Fig. 7D). In addition to the above-mentioned in vitro experiments, we also studied the effect of irisin on p53 acetylation in STZ-induced diabetic mice. We found that intraperitoneal injection of the SIRT1 inhibitor EX527 (10 mg/kg body weight, intraperitoneally injected, twice per day for 4 weeks) blocked the protective effect of irisin on cardiac function (Fig. 7E). Moreover, the irisin-mediated restoration of mitochondrial structure, reduction in left ventricular iron deposition (Fig. 7F and G), and reduction in p53 acetylation were blocked by the SIRT1 inhibitor, EX527 (Fig. 7H). Taken together, these data suggested that irisin could attenuate ferroptosis and protect cardiac function in DCM via the SIRT1-p53-SLC7A11/GPX4 pathway (Fig. 8).

## Discussion

Previous studies found that irisin inhibits myocardial apoptosis and fibrosis in mice with T2DM [15], but its protective effect on T1DM-induced cardiac remodeling remains unclear. Whether there is a change in circulating irisin level in patients with T1DM is controversial. In a study of 11 patients diagnosed with T1DM and 11 healthy controls, serum irisin levels were found to be higher in the T1DM group than in the control group [46]. By contrast, in a long-term study of 79 subjects with T1DM and 53 normal controls, plasma irisin levels were lower in subjects with T1DM than in controls [47]. Although the reasons for these discrepancies are not known, the method for the detection/quantification of irisin protein may be a critical factor [48], in addition to variables such as age, sex, and physical and treatment status. In our published study [49], we used four complementary methods, including western blot, immunohistochemistry, protein liquid chip assay, and liquid chromatography-tandem mass spectrometry (LC–MS), to quantify irisin in lung epithelial cells and tissues. In the present study, we used the same protocol and found that irisin levels in myocardial tissues, as well as in the serum, were reduced in mice with T1DM. Previous research reported that diabetes could impair AMPK-PGC-1α signaling in skeletal muscle, resulting in decreased PGC1-α transcription-dependent irisin levels, thereby reducing serum irisin levels [50–52].

Irisin exhibits versatile protective effects on organ injury, including pulmonary and cardiac ischemia/reperfusion injury [49, 53]. DCM is a serious complication of T1DM. In addition to skeletal muscle, irisin is also highly expressed and released from cardiomyocytes [18, 19]. Therefore, we analyzed further the expression of irisin in the myocardium. Interestingly, consistent with the finding of decreased serum irisin levels in STZ-induced diabetic mice, cardiac irisin protein expressions were also significantly decreased. Thus, we inferred that cardiac injury may be related to a low level of irisin in T1DM. Therefore, in this study, we investigated whether exogenous supplementation with irisin would protect against diabetic cardiomyopathy and found that irisin supplementation attenuates cardiac injury in T1DM mice by an anti-ferroptosis action, via SIRT1-mediated deacetylation of p53.

Previous studies have demonstrated that abnormal metabolism of Fe^2+^ not only increases the risk of insulin resistance and diabetes [54] but also causes cardiovascular diseases in diabetic subjects [7]. However, its underlying mechanism remains unclear. Ferroptosis is different from apoptosis in morphology, biochemistry, and genetics, and is defined as a Fe^2+^-dependent cell nonapoptotic cell death [55]. Previous studies found that ferroptosis is involved in myocardial remodeling in six-month-old db/db mice, and inhibition of ferroptosis by a ferroptosis-specific inhibitor prevents cardiomyocyte loss and cardiac dysfunction [56]. In the present study, we detected ferroptosis both in HG-treated H9C2 cells and in cardiac tissue of T1DM mice about 2 months after the onset of diabetes, as characterized by elevated Fe^2+^ content and lipid peroxidation levels, and decreased GSH levels and SLC7A11/GPX4 expressions, which were recovered by irisin treatment.

SLC7A11 (cystine/glutamate antiporter) is responsible for the uptake of extracellular cystine to supplement cellular cysteine, an essential biosynthesis precursor to GSH, that inhibits lipid peroxidation and ferroptosis [56]. We focused on the mechanisms underlying the upregulation of SLC7A11 after irisin treatment. The upstream regulators of SLC7A11 include p53, NRF2, ATF3, and BAP-1 [38–41]. Our data showed that p53 was the only candidate because HG increased p53 expression, which was reduced by irisin treatment. However, no significant change in the p53 mRNA level was observed. Therefore, we speculated that irisin might affect p53 post-transcription, especially its degradation. To test our hypothesis, H9C2 cells were treated with CHX, a protein synthesis inhibitor, and the p53 degradation rate was analyzed. We found that treatment with irisin promoted p53 degradation, thereby shortening its half-life. Among the post-translational modification is acetylation which regulates the stability of p53 protein [57]. The acetylation of p53 not only occupies the ubiquitination site of acetylated lysine residues but may also attenuate murine double minute 2 (MDM2)-mediated ubiquitination of other unacetylated lysine residues by inducing conformational changes in protein [58]. To determine whether irisin exerts its protective role by regulation of p53 expression, H9C2 cells exposed to HG were treated with irisin and found that irisin treatment significantly decreased p53 acetylation and p53 protein levels. As expected, after up-regulation of p53 expression by transfection of p53 plasmid in H9C2 cells, the inhibitory effect of irisin on ferroptosis was lost. Therefore, irisin decreases p53 acetylation and promotes p53 degradation to increase the expression of SLC7A11, consequently preventing the occurrence and development of ferroptosis.

Our results indicate that treatment with irisin did not affect p53 mRNA expression in HG-treated H9C2 cells, indicating that the irisin-mediated effect on p53 expression in HG-treated H9C2 cells could be due to post-transcriptional regulation. A variety of posttranslational modifications that can regulate p53 activity, including phosphorylation, acetylation, methylation, and sumoylation, have been described [59]. Acetylation of p53 on Lys382 (p53-K382Ac) was reported to play an important role in stabilization, nuclear localization, and transcriptional activation of p53 and can lead to p53 activation [60]. SIRT1, as an NAD + -dependent protein deacetylase, is the most extensively studied member of the sirtuin family; There are some studies about drug-induced stimulation or restoration of SIRT1 levels by exogenous administration and both of them are involved in the improvement of cardiac function in diabetic animals [61]. Interestingly, SIRT1 can regulate the acetylation of P53 at K382, and the latter one is the classical substrate of SIRT1 in multiple types of cells [44, 62, 63]. Aprevious study found that p53 deacetylation induced by SIRT1 may alleviate sepsis-induced renal injury by promoting renal tubular epithelial cell autophagy [64]. A recent study also showed that SIRT1 could inhibit ferroptosis-induced myocardial cell death through the p53 signaling pathway in myocardial ischemia/reperfusion injury [65]. Moreover, irisin can activate the SIRT1 signaling pathway in many tissues [45, 66]. Based on these aforementioned studies, we hypothesized that SIRT1 activation mediated the induction of p53 deacetylation after irisin treatment. Our results showed that HG downregulated SIRT1 protein expression but irisin treatment significantly upregulated its expression. Knockdown of SIRT1 by si-SIRT1 significantly inhibited irisin-induced p53 deacetylation and decreased p53 protein expression, indicating that irisin-mediated p53 inhibition may be mediated by SIRT1 activation. Additionally, the beneficial effects of irisin on DCM and ferroptosis were reversed by si-SIRT1 and the SIRT1 inhibitor EX527, which further indicated that irisin induced anti-ferroptosis and cardiac protection by activating the SIRT1 signaling pathway.

In conclusion, irisin protein is lower in the heart of T1DM mice; irisin supplementation ameliorates cardiac dysfunction in T1DM through an anti-ferroptosis action. Mechanistically, the upregulation of SIRT1 by irisin decreases protein of p53 expression and activity, consequently, induces SLC7A11 (cystine/glutamate antiporter) and GPX4 expressions and finally reduces ferroptosis. Irisin may be a promising therapeutic approach for T1DM-induced cardiomyopathy.

Nevertheless, some limitations must be considered for the interpretation of our study. First, it is widely known that the use of anesthesia can affect cardiac function. The influence of anesthetic agents on the ferroptosis process in T1DM in our study is unclear. Second, we showed that irisin protein was lower in serum from STZ-treated mice than controls, whether irisin is a biomarker for cardiac dysfunction need to be determined in the future. Finally, to investigate the molecular mechanisms underlying the protective effects of irisin against T1DM, we only focused on SIRT1. As a matter of fact, three major sirtuins associated with ferroptosis (SIRT1, SIRT3, and SIRT7) were monitored in cardiomyocytes [67–69], and the influence of irisin on the protein levels of SIRT3 and SIRT7 in our study is unclear, which also needed to be elucidated in the future.

### Supplementary Information

**Additional file 1: Figure S1.** Effects of irisin on general features in STZ-induced type 1 diabetic mice. **A** Comparison of body weight at different weeks (weeks 0, 1, 3, 5, and 7) in the indicated groups of mice (n = 8 per group). **B** Comparison of fasting blood glucose at different weeks (weeks 0, 1, 3, 5, and 7) in the indicated groups of mice (n = 8 per group). Data are expressed as the mean ± SD. One-way ANOVA, and Bonferroni’s post-hoc test. *P<0.05, ^**^P<0.01. *STZ* streptozotocin. **Figure S2.** Effect of irisin on the expression of SLC7A11 transcription factor in H9C2 cells. **A** Western blots (**A1**) and quantification (**A2**) of p53, ATF3, NRF2, and BAP-1 proteins in H9C2 cells after HG (35 mmol/L) exposure with or without irisin (10 nmol/L) for 24 h, All results were normalized to the expression level of GAPDH (n=4 per group). **B** Western blotting results (**B1**) and quantitative (**B2**) analysis of p53 expression in the heart tissue, All results were normalized to the expression level of GAPDH (n=4 per group). **C** qRT-PCR of the expression of p53 mRNA in H9C2 cells. (n = 4 per group). Data are expressed as the mean ± SD.One-way ANOVA, and Bonferroni’s post-hoc test. *P<0.05, ^**^P<0.01. *HG* high glucose, p53, tumor suppressor p53, *ATF3* activating transcription factor 3, *NRF2* nuclear factor erythroid-2 related factor 2, and BAP-1, breast cancer 1-associated protein 1. **Figure S3.** P53 overexpression and SIRT1 interference efficiency. **A** H9C2 cells were transfected with pcDNA3.1 (Vector) or p53 plasmids (0E-p53). The transfection efficiency of p53 plasmids in H9C2 cells was detected by Western blot. Representative Western blots (**A1**) with quantification (**A2**) showing p53 protein (n=4 per group). **B** Interference efficiency of siRNA-SIRT1 in H9C2 cells was detected by Western blot (n = 4 per group). Data are expressed as the mean ± SD. One-way ANOVA, and Bonferroni’s post-hoc test. *P<0.05, ^**^P<0.01. *HG* high glucose, *p53* tumor suppressor p53, and *SIRT1* Sirtuin 1. **Table S1.** Description of the primers used in this study. *p53* tumor suppressor p53, *GAPDH* glyceraldehyde-3-phosphate dehydrogenase.

## Data Availability

The data and methods involved in this study are available from the corresponding authors, and the involved materials are available from the relevant reagent vendors.

## Abbreviations

ATF3: Activating transcription factor 3
CCK8 Kit: Cell counting kit-8
BAP-1: BRCA1 associated protein 1
CHX: Cycloheximide
CK-MB: Creatine kinase-myocardial isoenzyme
DCM: Diabetic cardiomyopathy
DMEM: Dulbecco’s Modified Eagle Medium
DCFH-DA: 2, 7-Dichlorofluorescein diacetate
FBS: Fetal bovine serum
FNDC5: Fibronectin type III domain-containing protein 5
GSH: Reduced glutathione
GSSG: Oxidized glutathione
GPX4: Glutathione peroxidase 4
H&E: Hematoxylin and eosin
HG: High glucose
H9C2 cells: Rat cardiomyocyte cell line
LVEF: Left ventricular ejection fraction
LVFS: Left ventricular fractional shortening
LVIDs: Left ventricle internal diameter at end-systole
LVIDd: Left ventricle internal diameter at end-diastole
LPO: Lipid peroxidation
LDH: Lactate dehydrogenase
LC–MS: Liquid chromatography-tandem mass spectrometry
Masson: Masson’s trichrome
MDA: Malondialdehyde
MDM2: Murine double minute 2
MI/R: Ischemia/reperfusion
NC: Negative control
NRF2: Nuclear factor erythroid-2 related factor 2
PTM: Post-translational modification
PGC-1α: Peroxisome proliferator-activated receptor-γ coactivator 1-α
PBS: Phosphate-bufered saline
p53: Tumor suppressor p53
qRT-PCR: Quantitative real-time polymerase chain reaction
ROS: Reactive oxygen species
SD: Standard deviation
SDS-PAGE: Sodium dodecyl sulfate–polyacrylamide gel electrophoresis
SLC7A11: Solute carrier family 7 member 11
STZ: Streptozotocin
SIRT1: Sirtuin 1
TBST: Tris-buffered saline with Tween 20
TBS: Tris-buffered saline
T1DM: Type 1 diabetes mellitus
T2DM: Type 2 diabetes mellitus
TEM: Transmission electron microscopy
