# Supplementary material for: Irisin attenuates type 1 diabetic cardiomyopathy by anti-ferroptosis via SIRT1-mediated deacetylation of p53
Source: Cardiovasc Diabetol. 2024 Apr 2;23:116. doi: 10.1186/s12933-024-02183-5 (PMC10985893; doi:10.1186/s12933-024-02183-5)
Supplement: Supplementary file 1 — Additional file 1: Figure S1. Effects of irisin on general features in STZ-induced type 1 diabetic mice. A Comparison of body weight at different weeks (weeks 0, 1, 3, 5, and 7) in the indicated groups of mice (n = 8 per group). B Comparison of fasting blood glucose at different weeks (weeks 0, 1, 3, 5, and 7) in the indicated groups of mice (n = 8 per group). Data are expressed as the mean ± SD. One-way ANOVA, and Bonferroni’s post-hoc test. *P<0.05, **P<0.01. STZ streptozotocin. Figure S2. Effect of irisin on the expression of SLC7A11 transcription factor in H9C2 cells. A Western blots (A1) and quantification (A2) of p53, ATF3, NRF2, and BAP-1 proteins in H9C2 cells after HG (35 mmol/L) exposure with or without irisin (10 nmol/L) for 24 h, All results were normalized to the expression level of GAPDH (n=4 per group). B Western blotting results (B1) and quantitative (B2) analysis of p53 expression in the heart tissue, All results were normalized to the expression level of GAPDH (n=4 per group). C qRT-PCR of the expression of p53 mRNA in H9C2 cells. (n = 4 per group). Data are expressed as the mean ± SD.One-way ANOVA, and Bonferroni’s post-hoc test. *P<0.05, **P<0.01. HG high glucose, p53, tumor suppressor p53, ATF3 activating transcription factor 3, NRF2 nuclear factor erythroid-2 related factor 2, and BAP-1, breast cancer 1-associated protein 1. Figure S3. P53 overexpression and SIRT1 interference efficiency. A H9C2 cells were transfected with pcDNA3.1 (Vector) or p53 plasmids (0E-p53). The transfection efficiency of p53 plasmids in H9C2 cells was detected by Western blot. Representative Western blots (A1) with quantification (A2) showing p53 protein (n=4 per group). B Interference efficiency of siRNA-SIRT1 in H9C2 cells was detected by Western blot (n = 4 per group). Data are expressed as the mean ± SD. One-way ANOVA, and Bonferroni’s post-hoc test. *P<0.05, **P<0.01. HG high glucose, p53 tumor suppressor p53, and SIRT1 Sirtuin 1. Table S1. Description of the prim [file 12933_2024_2183_MOESM1_ESM.docx]

**Additional file 1:**

**
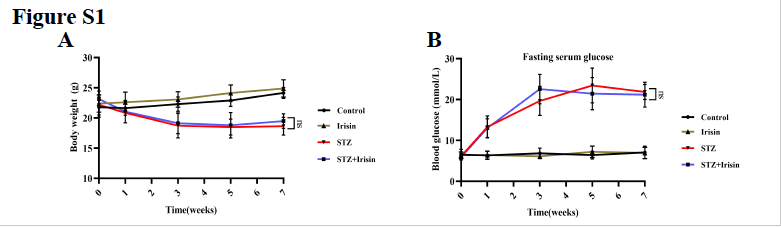
**

**Figure S1. Effects of irisin on general features in STZ-induced type 1 diabetic mice.**

**A.** Comparison of body weight at different weeks (weeks 0, 1, 3, 5, and 7) in the indicated groups of mice (n = 8 per group). **B.** Comparison of fasting blood glucose at different weeks (weeks 0, 1, 3, 5, and 7) in the indicated groups of mice (n = 8 per group). Data are expressed as the mean ± SD. One-way ANOVA, and Bonferroni’s post-hoc test. ^*^*P*<0.05, ^**^*P*<0.01. STZ, streptozotocin.


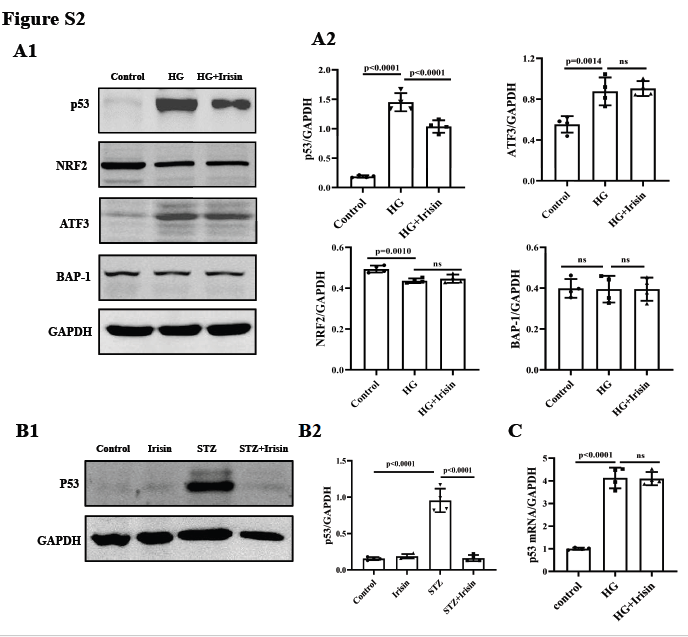


**Figure S2. Effect of irisin on the expression of SLC7A11 transcription factor in H9C2 cells.**

**A**. Western blots (**A1**) and quantification (**A2**) of p53, ATF3, NRF2, and BAP-1 proteins in H9C2 cells after HG (35 mmol/L) exposure with or without irisin (10 nmol/L) for 24 hrs, All results were normalized to the expression level of GAPDH (n=4 per group). **B**. Western blotting results (**B1**) and quantitative (**B2**) analysis of p53 expression in the heart tissue, All results were normalized to the expression level of GAPDH (n=4 per group). **C.** qRT-PCR of the expression of p53 mRNA in H9C2 cells. (n = 4 per group). Data are expressed as the mean ± SD.One-way ANOVA, and Bonferroni’s post-hoc test. ^*^*P*<0.05, ^**^*P*<0.01. HG, high glucose; p53, tumor suppressor p53; ATF3, activating transcription factor 3; NRF2, nuclear factor erythroid-2 related factor 2, and BAP-1, breast cancer 1-associated protein 1.

**
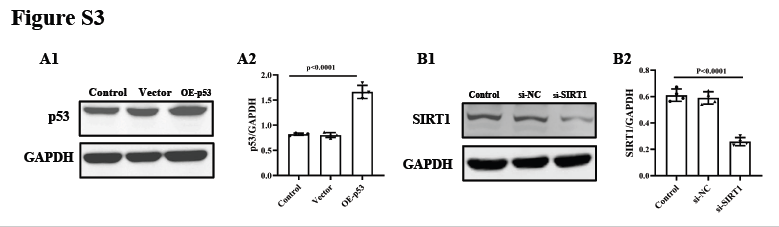
**

**Figure S3. P53 overexpression and SIRT1 interference efficiency**.

**A**, H9C2 cells were transfected with pcDNA3.1 (Vector) or p53 plasmids (0E-p53). The transfection efficiency of p53 plasmids in H9C2 cells was detected by Western blot. Representative Western blots (**A1**) with quantification (**A2**) showing p53 protein (n=4 per group). **B**, Interference efficiency of siRNA-SIRT1 in H9C2 cells was detected by Western blot (n = 4 per group). Data are expressed as the mean ± SD. One-way ANOVA, and Bonferroni’s post-hoc test. ^*^*P*<0.05, ^**^*P*<0.01. HG, high glucose; p53, tumor suppressor p53; and SIRT1, Sirtuin 1.

**Table S1. Description of the primers used in this study.** p53: tumor suppressor p53; GAPDH:glyceraldehyde-3-phosphate dehydrogenase.

Supplemental table S1: Primer List

| **#** | **Names of Primer** | **Sequence of Primer** |
| --- | --- | --- |
| 1 | *p53 (rat)Forward* | 5’- CCCCTGAAGACTGGATAACTGT -3’ |
| 2 | *p53 (rat)Reverse* | 5’- ATTAGGTGACCCTGTCGCTG -3’ |
| 3 | *GAPDH(rat) Forward* | 5’- CCTGCACCACCAACTGCTTA -3’ |
| 4 | *GAPDH (rat) Reverse* | 5’- GGCCATCCACAGTCTTCTGA -3’ |
